# The Contribution of Moral Case Deliberation to Teaching RCR to PhD Students

**DOI:** 10.1007/s11948-023-00431-7

**Published:** 2023-03-01

**Authors:** Giulia Inguaggiato, Krishma Labib, Natalie Evans, Fenneke Blom, Lex Bouter, Guy Widdershoven

**Affiliations:** 1 Department of Ethics, Law & Humanities, Amsterdam Public Health Institute, Amsterdam UMC Location Vrije Universiteit Amsterdam, De Boelelaan 1117, 1081HV Amsterdam, the Netherlands; 2 Department of Epidemiology and Data Science, Amsterdam University Medical Centers Location Vrije Universiteit Amsterdam, De Boelelaan 1117, 1081HV Amsterdam, the Netherlands; 3 Department of Philosophy, Faculty of Humanities, Vrije Universiteit, De Boelelaan 1105, 1081 HV Amsterdam, The Netherlands

**Keywords:** Responsible conduct of research, Doctoral training, Dialogical skills, Moral dilemmas, Research integrity, Moral case deliberation

## Abstract

Teaching responsible conduct of research (RCR) to PhD students is crucial for fostering responsible research practice. In this paper, we show how the use of Moral Case Deliberation—a case reflection method used in the Amsterdam UMC RCR PhD course—is particularity valuable to address three goals of RCR education: (1) making students aware of, and internalize, RCR principles and values, (2) supporting reflection on good conduct in personal daily practice, and (3) developing students’ dialogical attitude and skills so that they can deliberate on RCR issues when they arise. What makes this method relevant for RCR education is the focus on values and personal motivations, the structured reflection on real experiences and dilemmas and the cultivation of participants’ dialogical skills. During these structured conversations, students reflect on the personal motives that drive them to adhere to the principles of good science, thereby building connections between those principles and their personal values and motives. Moreover, by exploring personal questions and dilemmas related to RCR, they learn how to address these with colleagues and supervisors. The reflection on personal experiences with RCR issues and questions combined with the study of relevant normative frameworks, support students to act responsibly and to pursue RCR in their day-to-day research practice in spite of difficulties and external constraints.

## Introduction

Fostering responsible conduct of research (RCR) is increasingly important in the current research environment. RCR education has been identified as a crucial means to foster good practices and produce valid and trustworthy research (Forsberg et al., 2018; Resnik & Shamoo, 2011). Since the late 80s, RCR training programs have been developed around the world (Kalichman, 2013); they show diversity in goals, methodologies, process, and content (Marusic et al., 2016; Mulhearn et al., 2017) but they mostly aim at reducing research misconduct by fostering knowledge of guidelines and regulations and helping researchers make responsible choices (The National Academies of Sciences, Engineering and Medicine 2017; Shephard et al., 2015; Steneck & Bulger, 2007).

An important target group for RCR training programs is PhD students. Teaching PhD provides the opportunity to train the future generation of researchers. However, trainers might risk losing PhD students’ interest if they do not listen to, and constructively discuss, the difficulties they encounter in their own research environment.

Despite the growing literature on RCR teaching, and the positive results shown by some studies about the benefits of RCR education (Watts et al., 2017), there is little evidence on the effectiveness of RCR training in the long term (Anderson et al., 2007; Antes et al., 2010; Marusic et al., 2016; Mulhearn et al., 2017; Mumford et al., 2015). This scarcity of evidence is complicated by the fact that there is currently little consensus on what the goals of RCR training should be, which makes evaluation of effectiveness difficult (Mumford et al., 2015). In this paper we agree with those who argue that RCR trainings should help participants understand *why* RCR is crucial, *why* it is important to make RCR a topic of conversation, and *how* they can approach RCR issues when they arise (Eisen & Berry, 2002; Kalichman, 2014). Based on the RCR education literature, we can formulate the following three goals for RCR education targeted at PhD students: (1) make students aware of, and internalize, RCR principles and values, (2) support reflection on good conduct in personal daily practice (3) develop students’ dialogical skills so that they can deliberate on RCR issues when they arise. We argue that moral case deliberation – a case reflection method we use in the Amsterdam UMC RCR PhD course contributes to achieving these core goals. In particular we argue that the dilemma method (Stolper et al., 2016), a specific moral case deliberation method, which has been used now for almost twenty years in different contexts such as health care, education and research (Haan et al., 2018; Spijkerboer et al., 2019; Weidema et al., 2013), can, in synergy with other elements of RCR education, support the internalization of RCR values, provide a framework to reflect on personal experiences and dilemmas related to RCR and provide knowledge and skills required to engage in a dialogue about RCR. This specific method, which provides a structured approach to facilitate a group reflection while fostering a dialogical attitude, has not been used in RCR education previously.

In this paper, we describe the use of MCD in a broader PhD course that includes online individual learning, interactive lectures, working groups and other assignments (e.g., a mandatory meeting with PhD supervisors to discuss RCR issues). We present this course in its entirety as an example of how MCD could be integrated into broader interventions.

## Three Suitable Goals of RCR Education for PhD Students

A particular problem in RCR education, is that PhD students might score highly on their knowledge of rules but may still doubt their practical applicability or lack the motivation to adhere to them (Shephard et al., 2015). Guidelines and regulations communicated in RCR courses can conflict with the common practices or hierarchical dynamics in which PhD students operate, a tension which might lead to cynicism or moral distress (Geller et al., 2010; Resnik, 2016). Focusing on RCR issues could even be detrimental if trainees learn ‘what they can get away with’ or become disillusioned with the current system (Antes et al., 2010), both of which can lower the bar for engaging in questionable research practices. RCR is often perceived as something extrinsic, a set of externally imposed rules, a “regulatory burden” (The National Academies of Sciences, Engineering and Medicine 2017, p. 172) which researchers have to comply with. A strong focus on compliance with RCR rules leads to the creation of a “police state” (Pennock & O’Rourke, 2017, p. 244), where researchers perceive RCR as an obstacle instead of something intrinsic to the practice of research. Acting with integrity can be challenging for an early career researcher, not only because of their low place in the team hierarchy, poor mentoring, or career pressure, but also because ‘doing the right thing’ often requires more than simply applying a set of norms and guidelines (Payne, 2000; Zwart & Ter Meulen, 2019). Guidance does not cover all situations researchers find themselves in and needs interpretation in specific contexts.

Instead of focusing merely on identifying and sanctioning detrimental practices, RCR education should focus on promoting desirable behaviors and a culture in which the intrinsic values (i.e., beliefs and ideals motivating actions) and norms (i.e., action guiding rules) of research are discussed and put forward (Ferguson et al., 2007; Kalichman, 2014). This includes encouraging and empowering PhD students to engage in open conversations with their colleagues and supervisors about the ethical dimensions of research. Based on growing consensus within the existing literature we formulate three suitable goals for RCR training. Below we will elaborate on each of these goals.

- Make students aware of, and internalize, RCR principles and values.

RCR guidelines and codes of conducts are an important element of any RCR course. However, PhD students need to feel motivated to support RCR, not because an external expert says so, but because they understand that to develop trustworthy knowledge and to serve society adequately, research has to be done with integrity (Payne, 2000; Shephard et al., 2015). As Pennock and O’Rourke (2017) argue, few RCR education programs investigate the fundamental set of values which are intrinsic to the realm of research. A strong emphasis on rules, if not contextualized and internalized by PhD students, can feed the dangerous idea that research integrity norms hamper the pursuit of personal goals (Shephard et al., 2015). When teaching RCR to PhD students, it is therefore important “to arm them with a positive disposition toward RCR, with a sense that there are things they can do in the face of concerns, and with a belief that they are part of a culture that takes RCR seriously” (Kalichman, 2014, p. 70). Reflecting on their personal and professional motivation, values, and moral character fosters the internalization of the principles of good science and enables students to link reflections on RCR to their professional development. This reflection also highlights the relationship between RCR principles and personal values and goals, as well as the possible tensions between the two (Resnik, 2012).

(b) Supporting reflection on good conduct in personal daily practice.

PhD students should be supported to relate RCR concepts and rules to their own personal experiences by addressing the questions that they have to deal with in their everyday research practice (Löfström, 2012). Reflection on one’s personal experiences is crucial for fostering application of RCR principles and regulations but also for acknowledging possible systemic constraints which might work against RCR in students’ own research environment. Trainees need to reflect on the meaning of RCR values for their personal research practice, recognizing possible tensions between values (e.g., accountability and respect for their supervisors’ authority) and potential issues which might prevent them to act with integrity, in order to reflect together on how these can be dealt with in their day-to-day work (Shephard et al., 2015; Hyytinen & Löfström, 2017).

(c) Developing students’ dialogical skills so that they can deliberate on RCR issues when they arise.

As researchers at the beginning of their careers, PhD students go through a process of acculturation to research practice. They are particularly influenced by the culture of departments or research groups that they belong to (Joynson & Leyser, 2015) and often depend on their supervisors and mentors to ensure responsible research practices (Titus & Ballou, 2014). Unfortunately, PhD students rarely discuss RCR issues with their supervisors and colleagues (Alfredo and Hart 2011; Eisen & Berry, 2002; Plemmons & Kalichman, 2018). Fostering dialogue and exchange between PhD students, colleagues, and supervisors, can provide PhD students with the opportunity to engage in conversations about complex issues while being open to hearing and learning from other’s perspectives. Learning how to engage in a process of shared reflection on RCR issues can encourage PhD students to speak up or to ask for support when dealing with RCR challenges and complements the more formal part of RCR education. This can, in turn, drive real change in the research climate in which PhD students operate (Anderson et al., 2007; Hooper et al., 2018).

We suggest that Moral Case Deliberation can help accomplish these goals. In what follows, we describe a RCR course for PhD students which incorporates MCD.

## Embedding the Core Goals in Educational Practice: The Role of Moral Case Deliberation within the RCR Course at Amsterdam UMC

### The Amsterdam UMC RCR PhD Course

The Amsterdam UMC has developed a blended learning course for PhD students which consists of three parts. The course consists of an online module, two face-to-face sessions, and an evaluation (Box 1). Participants devote an estimated 56 h in total to the course.

**Box 1:** Training structure

1. Preparation (20 h)
Online module by Epigeum^a^
Writing a description of an RCR issue they encountered personally
2. Face-to-face sessions (16 h over 2 non-consecutive days)
Interactive lectures (including Rotterdam Dilemma Game^b^ or fiction movies fragments)
2 Workgroups: 1) Confessing errors in research; 2) Research culture
2 MCD sessions (of 2 h each)
3. Evaluation (20 h)
Writing a self-reflection on the content of the course
Discussion with own supervisors about RI topics and writing a report of the meeting

^a^Available at: https://www.epigeum.com/courses/research/research-integrity/.

^b^Available at: https://www.eur.nl/en/about-eur/policy-and-regulations/integrity/research-integrity/dilemma-game.

The blended learning approach is aimed at balancing individual learning, which can be stimulated online, with group interaction (Mulhearn et al., 2017; Hull et al. 1994). Online courses alone do not give participants the chance to interact and engage in group reflection processes (Aggarwal et al., 2011) but have been proven effective in conveying concepts and instructions which require individual processing (Todd et al., 2017). In the Amsterdam UMC course, we use an online course to introduce important topics related to RCR. By taking the online module, participants learn about core RCR concepts and issues such as: professional responsibility for good research practices; RCR principles; desirable responses to research misconduct; research design (including research with human participants and research with animals); publication, and responsibility to society. The online module allows students to acquire a common entry level knowledge and vocabulary which will enable them to engage in fruitful and more in-depth interactive learning during the face-to-face sessions.

The two face-to-face days are composed of plenary participatory lectures, working groups, and MCD sessions (Box 1). During the interactive lectures, trainers build on the content of the on-line training by providing an opportunity for participants to ask questions, and by providing more in-depth explanations of the most difficult topics. By using tools such as the Dilemma Game developed by the Erasmus University Rotterdam, movie fragments and illustrations, trainers foster reflection on how things could go wrong or right in real day-to-day situations which PhD students are likely to experience themselves; this allows them to apply the knowledge they have acquired in the online module in an interactive way.

In the working groups, participants engage in a group reflection (of approximately 10 to 15 people) on real cases about research culture or errors in research. This shared reflection process encourages PhD students to realize how the research system and its interaction with research culture and individual factors can influence researchers’ choices. Also, during each face-to-face training day, trainees take part in an MCD session. During these sessions, participants jointly reflect on a moral dilemma experienced by one of them^1^ and selected by the group and by the teacher, following a structured conversation method, guided by a trained facilitator (Stolper et al., 2016).

After participating in the course, PhD students are asked to write a personal reflection about what they have learned during the course and how these insights can be implemented in their own research practice. Additionally, students organize a meeting with their supervisors to discuss the written reflection jointly. The goal of this assignment is to motivate PhD students to have a dialogue about RCR with their supervisors and to link what they have learned during the course with their own research context and culture. After the meeting, students submit their reflection and a report summarizing the content of the discussion with the supervisor.

### RCR Education Core Goals: The Contribution of MCD

MCD is a structured dialogue, aimed at exploring the moral dimension of the specific case by investigating values, perspectives and emotions (Weidema et al., 2013). This method is widely used in the Netherlands in different contexts, especially in health care institutions, but is also adopted in research and education (Molewijk et al., 2008; Spijkerboer et al., 2019; Stolper et al., 2012; van der Dam et al., 2011). During MCD sessions, participants are invited to focus on real experiences and reflect on personal values and concerns. The values and moral intuitions of those involved in the case are reflected on by assuming a dialogical attitude (Metselaar et al., 2015b; Widdershoven & Metselaar 2012). Inspired by Gadamer’s notion of dialogue (2004), during MCD, participants are invited to postpone their judgements and have open attitude, showing curiosity toward their own and others’ point of view. Participants express their own ideas to exchange perspectives and learn from others, instead of convincing them of their own views. Supported by the facilitator, they are invited to address others by asking factual and open questions.

The process consists of nine steps and is guided by a certified MCD facilitator, who has received intensive training in moderating dialogical processes of shared reflection. After an introduction to the method (step 1) the case presenter is invited to introduce the case (step 2). Then the facilitator guides the case presenter and the group in formulating the dilemma at stake (step 3). The moral dimension of the case is explored and participants are asked to reflect on what possible negative consequences could follow from the two alternative courses of action delineated in the dilemma. After a round of questions to help participants clarify any details about the case (step 4), participants are asked to analyze the case from the perspective of all the stakeholders involved and to reflect on (a) which values are important for the stakeholders in the case, and (b) which norms (i.e., rules of action) would allow them to act upon those values (step 5). The facilitator writes the result of this process on a whiteboard, creating an overview of perspectives (i.e., stakeholders), values, and norms (see Box 2 for an example). This allows participants to compare the different perspectives, and see differences and similarities between them. After looking for alternative actions, which might represent a possible solution to the dilemma (step 6), participants are invited to make a choice for themselves and decide (a) what course of action they would support, (b) motivated by which values, (c) what value would be harmed by the action, i.e., what would be the negative consequences of the preferred course of action, and (d) what they would do to address these potential negative consequences (step 7). This is followed by a dialogue led by the facilitator about differences and similarities between individual choices, in order to jointly investigate which values are important in dealing with the situation (step 8). The session ends with formulating action points and evaluating the process (step 9). In the following, we explain how MCD can help RCR trainers in addressing the three core goals of RCR training described earlier.

#### Raising Awareness and Cultivating Internalization of Values Concerning RCR

Case reflection has proven to be an effective tool for building bridges between theories and practices and encouraging active learning processes (Bagdasarov et al., 2013; Jones et al., 2010; Mumford et al., 2009). In the Amsterdam UMC course, students experience two MCD sessions as part of their PhD course. During these two sessions they engage in conversations about their own RCR dilemmas, reflect on what RCR means to them personally, and jointly investigate moral dilemmas involved. This comes indeed with some risks, as personal experiences of a sensitive kind are shared. These risks can be mitigated in different ways. First, the facilitator explains to the group that the case will be investigated in a dialogical way, which implies openness and not judging the other, but asking questions to get to know the other’s perspective. The facilitator also takes care that participants keep to these rules, and makes sure that the process is conducted in a in a sensitive and respectful way in order to protect the case presenter from possible emotional burden. Second, students are instructed not to share names or details that could make the people involved in the case directly identifiable and are required to keep the confidentiality of everything said during the MCD session. Third, the facilitator receives the students’ cases in advance and can therefore pre-select the ones that are more suitable to be discussed in class. Fourth, if a case of misconduct becomes apparent the facilitator can approach the student and advise them regarding the procedures and support they can get in addressing the issue at stake. In general, the cases brought up by students do not regard right out misconduct, but questionable research practices, which are suitable for reflection in groups.

During the session, the facilitator invites PhD students to reflect on the values of all the stakeholders involved, (i.e., student, supervisor, collaborator, editor, etc.), and asks them to put themselves in the case presenter’s shoes to consider how to act in the situation presented by one of their colleagues. Next to identifying values, the participants are invited to reflect on what is needed to transform those values into actions by asking themselves “What should be done to act upon this value in this situation?” e.g. “What should I do to act upon e.g., ‘honesty’ in this situation?”. Below a table example is presented (Box 2).

**Box 2:** Case example

CASE:
My supervisor offers me to take over a fellow PhD student’s paper, which is almost finished, as first author. This colleague cannot work on it anymore and I need a publication.
Should I accept or should I refuse?

Reflecting on this case leads to the following table		
Perspectives	Values	Norms
Case owner (PhD student)	Accountability	I should build upon my own work
	Ambition	I should publish as soon as possible
	Obedience	I should not go against my supervisor
	Honesty	I should not take credit for someone else’s work
Supervisor	Accountability	I should accomplish my task as supervisor
	Publication	I should not waste the efforts made so far and I should ensure that results are published
Fellow PhD student (original author)	Ambition	I should be first author
	Support	I need help to get this published
	Respect	I should preserve a good relationship with my supervisor

Being asked to look at the situation from different perspectives, students are able to acknowledge distinct motivations and possible conflicts between them. While outlining and reflecting on perspectives and values, students realize that the participants in the case do not just follow rules, but act from internal motivations. Through this process of shared reflection, students understand what principles are relevant for those involved in the situation and, by putting themselves in the shoes of the case presenter, also become aware that values (or value orientations) relate to internal motivations and views on what is important in one’s work. Participants’ suggestions sometimes represent ‘value orientations’ and not necessarily ‘values’ by definition. Often values such as “honesty” or “transparency”, which can be found in most codes of conduct (e.g., the European Code of Conduct for Research Integrity) are mentioned (ALLEA All European Academies 2017). However, also less virtuous values such as “ambition”, “publication” and “respect for authority” can be mentioned by participants, as is seen in the case example.

During the MCD session, the internal motivations and moral compass that students have are made explicit and become the object of shared reflection. Making values explicit supports participants in understanding what is important for them as individuals and as professionals in a specific situation. While jointly discussing their values, students become aware of how principles and values which are essential for RCR relate to their internal moral compass, as well as what it means to act upon them in a specific situation. Thus, the goal of the MCD session is not necessarily to find an agreement on how to act in the situation at stake but primarily to jointly cultivate students’ own moral competence and character through dialogue. Thanks to the process of shared self-reflection, which culminates in step 7 and 8, where students have to make their own choice about the case and justify it on the basis of the values they consider important (see Box 3), participants cultivate their moral characters, and reflect on what it means for them to be a ‘good’ researcher. This process fosters reflection on what students deem important and encourages trainees to see values involved in RCR as personal and aspirational, while fostering their internalization.

#### Supporting Students to Reflect on Personal Daily Practices Related to RCR

During the MCD, PhD students reflect on a real case brought up by one of their colleagues and selected by the students themselves. When first asked to share a case of their own, i.e. a situation in which they experienced a research integrity dilemma, students often reply that they do not have any. Sometimes, participants think that challenges they experience in their work do not consist of an actual *moral* dilemma because there is a ‘clearly correct way to act in theory’, but it’s just ‘that practice is different from theory’. However, inspired by their peers and guided by the facilitator, they realize that RCR questions and dilemmas do occur in their work but are often unnoticed, unacknowledged or not reflected upon. The MCD approach allows students to recognize that these moments of doubt do have a moral dimension because they involve conflict of (sometimes equally) important values: e.g. collaboration vs. honesty (about contributions). By encouraging students to share their questions and dilemmas, and jointly think about how to address them while acknowledging possible constraints, such as hierarchy, personal ambitions, or conflict with colleagues, MCD provides students with the tools and the moral vocabulary (e.g., values, conflict, norms, perspectives) to talk about dilemmas with others while engaging in a shared self-reflection process about their daily experience with RCR issues.

After the reflection on the values and norms table (see Box 2), participants are asked to make their own decision about the case and think about: (a) what they would do in the situation of the case presenter, (b) which value or principle would guide their decision, (c) what values would be harmed, or what would be the potential negative consequence of the selected action and (d) what they would need specifically to act upon their decision, also in terms of mitigating any potential damage their decision might cause. An example of this, following from the case described in Box 2, is reported here below, showing the choices of three participants (Box 3).

**Box 3:** Reflection on possible courses of action

Participant	Choice of action	Because of …	In spite of potentially damaging …	How do I minimize the harm caused?	What do I need?
Student 1	I accept first authorship of the paper	Ambition	Honesty	Talk to my supervisor and to the other authors and express my doubts	Courage
Student 2	I accept first authorship of the paper, but take more time to study the material to see if I can really contribute	Honesty	Ambition	Confront my supervisor and set conditions for my contribution	Time
Student 3	I refuse first authorship	Accountability	Career	I offer to look at the data and assist the colleague who originally started the work on the paper	Support from my supervisor

In this step, students have to reflect on what intrinsic motivation, or value, would move them to action while acknowledging what possible negative consequences might follow from their choice. In this way, the consequences of their actions, which in daily practice often represent a constraint for students to act upon their own RCR values, are acknowledged, and possible ways to mitigate the damage that could follow from them is discussed. For instance, trainees can mention that acting upon the value of honesty would damage the relationship with a supervisor and go against the research culture of their research group. They then might come up with solutions to minimize the negative consequences they foresee, for instance by deciding to include the second supervisor or a third (senior) colleague in the conversation, or asking a confidential research integrity counsellor for advice.

Thinking about how to limit the damage caused by their choice empowers trainees to address uncertainties and constraints such as hierarchy and pressure in a constructive and practical way. Trainees not only acknowledge these constraints with their peers but are also asked to reflect together on how to practically face them. They become aware that they need “courage”, “support” or “time” to act on their preferred choice, as can be seen in the case example. They give each other peer support by sharing strategies, good practices, and approaches to deal with specific problems. Voicing these needs makes choices more concrete and shows participants the possibilities for acting with integrity in their own research practice. Moreover, the MCD approach fosters awareness around personal questions and dilemmas while exploring personal values, perspectives and possible conflicts between them. This process can result in more considered actions and supports students to develop new moral habits and character which can support them when facing difficult situations. In the Amsterdam UMC course, students continue the process of reflecting on their personal daily practices while writing a self-reflection assignment on how the content of the course relates to their research environment and professional context. In this way, after having the chance to reflect on specific RCR cases in a group setting, students are asked to think more broadly about their practices and how these could relate to the content of the course.

#### Fostering the Acquisition of Dialogical Attitudes and Skills Necessary to Deliberate on RCR Issues

Key to the MCD process is the dialogical attitude that participants need to assume during the entire session. The facilitator starts the session by explaining the characteristics of a dialogue and asking participants to assume a dialogical and open attitude, led by curiosity. This requires willingness to put one’s own preconceptions and prejudgments aside, and be open to genuinely listen to others. The facilitator explains that the goal of MCD is not to find easy ways out of the dilemma, but to learn from each other by exploring the situation from different angles. This can be difficult, especially at the start. Students naturally try to offer solutions, or already have pre-formed ideas about what is right or wrong. However, practicing a dialogical attitude enables participants to shift the focus from the goal of solving the dilemma to the concerns that lie behind the moral question that generated it. This leads to a broadening of views, comparing different perspectives and becoming capable of stepping into someone else’s shoes to deepen the understanding of the issue at stake.

In order to do that, participants need dialogical skills, such as postponing judgments, asking open and factual questions, listening to the viewpoints of others, using a moral vocabulary and applying it to the situation, which in combination with a dialogical attitude can foster a process of mutual learning and shared reflection. The facilitator supports participants in developing a dialogical attitude and dialogical skills by demonstrating that attitude and skills themselves. For instance, in step 3 and 4, which entail formulating the dilemma and asking questions for clarification, participants tend to propose alternative solutions or provide advice on how to get out of the dilemma. Often, they formulate questions such as: “Couldn’t you solve this by doing A or B?”. This question is not an open and factual question since it implies a recommendation, and does not help in broadening the horizon of understanding but instead steers the conversation towards a possible solution. The facilitator can ask the group to reflect on the quality of such questions and thereby support them to develop a sensitivity towards the type of questions which are supportive of a dialogue conducive to mutual understanding. At the end of the session, some students mention that things seemed quite simple from the start, but after the joint analysis, the picture starts to become more complex and they understand others’ positions more.

In the above case example, participants are invited by the facilitator to compare their individual answers (step 8). They are asked not to argue for their own choice, but to understand the choices made by the others, and consider the relevance of the values and norms which others put forward for their own professional practice. This may result in a more open view on one’s own initial values and concerns. Rather than arguing for their own decision whether or not to accept first authorship, they come to see the relevance of alternative views, and to develop more subtle and balanced solutions. They come to see that simply taking over first authorship does not do justice to the work of the colleague who did most of the work; however, refusing first authorship might not be helpful, as the colleague who performed the research will not see the results published. Listening to the considerations and suggestions of other participants may result in finding new ways to deal with the situation, for example by discussing the options with the colleague who did the initial work.

After experiencing the process of jointly analyzing a real case and exercising dialogical attitudes and skills in the context of an MCD session, PhD students report feeling empowered to have open RCR conversations with their colleagues. The course also offers students the opportunity to further practice engaging in a dialogue about RCR through an assignment where they meet with their supervisor to discuss the content of the course in relation to their own research and work context. Discussing RCR with peers during the course and with their supervisors can empower students to discuss RCR issues in their daily practice and make them aware of being part of a bigger research community, to which they can appeal to when in doubt about what to do.

## Discussion and Conclusions

In this paper, we highlight three important goals of RCR education for PhD students, namely fostering students’ awareness and promoting the internalization of values related to RCR, supporting reflection on good conduct in real RCR issues, and engaging in dialogues about RCR with supervisors and peers. In particular, we have argued that MCD, a tool we use in our Amsterdam UMC RCR course for PhD students, has unique features and a specific theoretical background which make it particularly suitable to address these aforementioned goals and therefore provides RCR educators with a practical example of how these goals can be addressed in practice. To optimize the advantages of using MCD, we have integrated the tool into a course consisting of multiple complementary elements. Traditionally, RCR education has focused on providing trainees with knowledge of concepts, rules, regulations, and relevant skills, such as critical thinking (Kalichman, 2014; National Institute of Health, 1989; Plemmons & Kalichman, 2013). Of course, knowledge of RCR issues, and the skills needed to reason, are necessary to engage in responsible research practice. However, if participants lack the ability and motivation to reflect and deliberate on real dilemmas experienced in their daily practice, the value of possessing such knowledge and skills can be questioned (Clarkeburn, 2002). Various RCR courses aim to reduce research misconduct by affecting behavioral change in researchers (Kalichman, 2007, 2014). However, there is a lack of evidence which shows that RCR courses can significantly influence researchers’ behaviors (Anderson et al., 2007; Antes et al., 2010). It is unlikely that a single RCR course can have such an effect (Kalichman, 2014). Alternatively, courses that empower students to reflect and engage in dialogues with others can contribute towards building a larger culture of RCR, in which RCR issues become a joint reflective endeavor between researchers (Kalichman, 2013). Such an environment of joint reflection allows PhD students to build on insights gained during the course and apply them in their daily practice.

There are currently multiple tools available for RCR trainers who aim to stimulate students to internalize principles of RCR and reflect on them with others, including case study discussions (Macrina & Munro, 1995; Pimple, 2007), role playing (Brummel et al., 2010), videos and vignettes (American Association for the Advancement of Science n.d.; Vasgird, 2011) and games (Erasmus University Rotterdam n.d.). In particular, problem-based approaches are growing in popularity to improve practical application of theory and improve critical thinking (Fuerholzer et al., 2020; Jones et al., 2010). We believe that MCD can serve as an additional tool available to RCR trainers. What makes MCD unique is its focus on reflection on personal experiences and values related to research conduct and on developing dialogical skills potentially strengthening a professional ethic. Some authors have argued that using cases that are highly complex can overwhelm RCR trainees and hinder the learning process (Bagdasarov et al., 2013; Watts et al., 2017). Reflecting on a case in the context of MCD minimizes this risk due to the structured step-by-step approach and the focus on a real case experienced by one of the participants.

Currently, no data are available on the use of MCD in RCR education, but MCD is an established tool in the clinical setting aiming to increase moral competence, teamwork and action, and has also been evaluated in this context, showing effectiveness in relation to learning outcomes (Stolper et al. 2016; Haan et al., 2018; Snoo-Trimp et al., 2020a, 2020b). There is consistent evidence that in the field of healthcare, MCD is effective in enhancing professionals’ willingness and abilities to recognize ethical issues and to engage in conversations about them with their colleagues (Snoo-Trimp et al., 2020a, 2020b). MCD has been shown to provide participants with the tools necessary to successfully navigate personal cases and dilemmas (Stolper et al., 2016; de Snoo-Trimp et al., 2020a, 2020b). First, it makes participants postpone judgments, creating a safe space to discuss sensitive issues, as well as the possibility for co-creating strategies to overcome RCR issues and share advice and peer support (Inguaggiato et al., 2019). Second, it helps participants to obtain a clearer image of all relevant explicit and implicit values at play, in order to consider the possibilities of dealing with them (Metselaar et al., 2015a). Third, it links the normative elements of RCR (e.g., guidelines and regulations) to daily practice (Molewijket al. 2011b). Finally, since the role of the facilitator is to guide the process, rather than the content of the discussion, participants have ownership over insights gained during the reflection.

By inviting participants to make explicit values and norms, MCD helps participants to become aware of their own values, internal motivations, and views about research, thus providing a basis for internalization of principles of good research practice. Additionally, MCD allows participants to reflect on personal daily practices by focusing on a real-life case of one of the participants. Finally, MCD stimulates PhD students to practice dialogical skills, necessary for a constructive exchange with others about RCR issues. We recommend the use of MCD, combined with other interactive ways of teaching, in RCR courses aimed at motivating PhD students and providing them with the skills and knowledge to act responsibly in their day-to-day research practice. 

## Data Availability

Not applicable.

## Footnotes

1 Participants are asked to protect confidentiality and not to share any information about the discussed case.
